# Dynamin-Like Proteins Are Potentially Involved in Membrane Dynamics within Chloroplasts and Cyanobacteria

**DOI:** 10.3389/fpls.2018.00206

**Published:** 2018-02-22

**Authors:** Ruven Jilly, Nadir Zaman Khan, Henrik Aronsson, Dirk Schneider

**Affiliations:** ^1^Institute of Pharmacy and Biochemistry, Johannes Gutenberg University Mainz, Mainz, Germany; ^2^Department of Biotechnology, University of Malakand, Malakand, Pakistan; ^3^Department of Biological and Environmental Sciences, University of Gothenburg, Gothenburg, Sweden

**Keywords:** dynamin, thylakoid membrane, membrane fusion, membrane biogenesis, cyanobacteria, chloroplasts

## Abstract

Dynamin-like proteins (DLPs) are a family of membrane-active proteins with low sequence identity. The proteins operate in different organelles in eukaryotic cells, where they trigger vesicle formation, membrane fusion, or organelle division. As discussed here, representatives of this protein family have also been identified in chloroplasts and DLPs are very common in cyanobacteria. Since cyanobacteria and chloroplasts, an organelle of bacterial origin, have similar internal membrane systems, we suggest that DLPs are involved in membrane dynamics in cyanobacteria and chloroplasts. Here, we discuss the features and activities of DLPs with a focus on their potential presence and activity in chloroplasts and cyanobacteria.

## Chloroplasts and Cyanobacteria Contain Two Inner Membrane Systems

Cyanobacteria and eukaryotic chloroplasts are evolutionary deeply connected, as primordial eukaryotic organisms had engulfed cyanobacterial ancestors in an endosymbiotic event. Incorporation of the bacteria into the cell metabolism finally resulted in development of a new organelle, the chloroplast, and the first oxygenic photosynthetic eukaryotes arose (reviewed in greater detail in Hohmann-Marriott and Blankenship, 2011; Jensen and Leister, 2014). During the course of evolution, several metabolic functions were lost in this newly developed organelle, and many genes of cyanobacterial origin were transferred from the new organelle into the genome of the host eukaryote (Martin and Herrmann, 1998; Martin et al., 2002; Kleine et al., 2009). Nevertheless, the ultrastructure of cyanobacteria and chloroplasts is still very similar, and the process of oxygenic photosynthesis as well as the proteins and cofactors involved therein are largely conserved (Hohmann-Marriott and Blankenship, 2011). In both cyanobacteria and chloroplasts, the components of the photosynthetic electron transport chain are localized within a specialized and unique internal membrane system, the thylakoid membranes (TMs). While the TM system is a completely separated and enclosed membrane system in chloroplasts and cyanobacteria, the exact fine structure of TMs can differ. In chloroplasts, TMs typically form multiple membrane stacks, which are connected by unstacked TMs, called grana and stroma lamellae, respectively (Adam et al., 2011), whereas TMs are organized in a sheet-like structure in cyanobacterial cells. However, the detailed TM arrangement can vary significantly in cyanobacterial strains (Herrero and Flores, 2008).

The structure of the TM system is highly dynamic in plant chloroplasts, and the amounts as well as the subcellular organization of TMs alter in response to changing environmental conditions (Chuartzman et al., 2008; Kirchhoff et al., 2011; Herbstova et al., 2012; Iwai et al., 2015). It has been stated that the dynamics observed in plant chloroplasts can essentially only be explained by the existence of a protein machinery that controls membrane fission and fusion processes (Chuartzman et al., 2008; Kirchhoff et al., 2011; Herbstova et al., 2012), analogous to the machineries involved in fusion of other organelle membranes, such as the ER, the Golgi apparatus, and the mitochondria (e.g., reviewed by Bonifacino, 2014). In fact, membrane connections and membrane fusion/fission processes have already been discussed for a long time (Nickelsen et al., 2011; Karim and Aronsson, 2014; Rast et al., 2015), but experimental evidence still is scarce. While described in chloroplasts, TM dynamics are essentially not studied in cyanobacteria yet. In cyanobacteria, individual TM layers appear to be as interconnected as in chloroplasts, and individual TM layers fuse and form “holes,” which are discussed to be required for intracellular transport (Nevo et al., 2007; Liberton et al., 2011a,b). Thus, the structure of TMs is most likely as dynamic in cyanobacteria as in chloroplasts. However, in contrast to chloroplasts, where TMs potentially develop *de novo* from the chloroplast inner envelope membrane (Muehlethaler and Frey-Wyssling, 1959; Morré et al., 1991), in cyanobacteria TMs do probably not form *de novo* but assemble from existing structures (Barthel et al., 2013).

## Dynamin(-Like) Proteins Are Involved in Membrane Remodeling Processes in Prokaryotes and Eukaryotes

**FIGURE 1:**
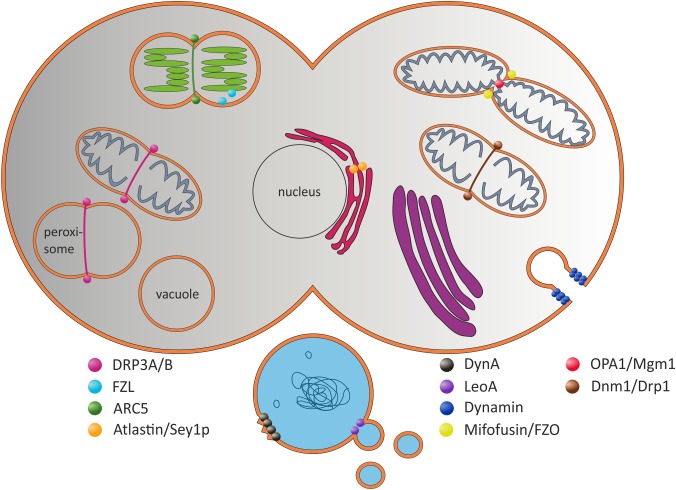
Selected (B)DLPs and their proposed *in vivo* functions. The prototypical Dyn protein is involved in the formation of clathrin-coated vesicles at the plasma membrane (Hinshaw and Schmid, 1995). Dnm1-like proteins are involved in mitochondrial scission. ARC5 is involved in chloroplast division (Smirnova et al., 2001; Gao et al., 2003; Mears et al., 2011). Atlastin/Sey1p are potentially involved in ER fusion, whereas Mitofusin/FZO fuse the inner and OPA1/Mgm1 the outer mitochondrial membranes (Hermann et al., 1998; Santel and Fuller, 2001; Frezza et al., 2006; Meeusen et al., 2006; Hu et al., 2009; Orso et al., 2010). The plant FZL protein is localized at the chloroplast inner envelope and the TM (Gao et al., 2006). In prokaryotic cells, DynA is proposed to be involved in membrane protection and/or membrane repair, and LeoA has been suggested being a component of a vesicle release system (Michie et al., 2014; Sawant et al., 2016).

Dynamin-like proteins (DLPs) [also called, dynamin-related proteins (DRPs)] are involved in diverse membrane-related processes in prokaryotic and eukaryotic cells, involving membrane fusion, membrane scission, membrane protection, and/or membrane stabilization (**Figure 1**). DLPs are members of a protein superfamily of GTPases. To separate DLPs from other GTPases, such as Ras-like GTPases involved in signal transduction, proliferation and survival of cells, DLPs are also entitled “large GTPases” (Praefcke and McMahon, 2004; Lu et al., 2016). The first identified member of the dynamin family, MxA and its yeast homolog Vps1 were found being involved in virus resistance, vacuolar protein sorting, fission of endosomal membranes and in endocytic events (Staeheli et al., 1986; Smaczynska-de Rooij et al., 2010; Chi et al., 2014). Simultaneously, the founder of the dynamin superfamily, the prototypical dynamin protein (Dyn), was shown to be crucial for the scission of clathrin-coated endocytic vesicles from eukaryotic plasma membranes (Shpetner and Vallee, 1989; Hinshaw and Schmid, 1995). Upon triggering membrane fission, MxA and Dyn form dimers that interacts head to tail, resulting in formation of inactive tetramers. In presence of lipids, the structure reorganizes resulting in rearrangement of this auto-inhibitory structure, and the GTP hydrolysis rate of Dyn increases from 2.6 to 105 min^-1^ (Song et al., 2004; Reubold et al., 2015). The mechanistic details of this GTP-driven process are described in more detail elsewhere (Praefcke and McMahon, 2004; Antonny et al., 2016). However, besides the prototypical Dyn protein, a group of related DLPs is active in/at different eukaryotic organelles. E.g., the *Drosophila melanogaster* DLP Atlastin and its yeast counterpart Sey1p are required for fusion of ER membranes (Hu et al., 2009; Orso et al., 2010), and the yeast protein Dnm1 and its mammalian homolog Drp1 are involved in mitochondria scission (Smirnova et al., 2001; Legesse-Miller et al., 2003; Ingerman et al., 2005; Mears et al., 2011; Ugarte-Uribe et al., 2014). Similar to Dyn, the GTPase activity of Drp1 also increases in presence of lipids (Bustillo-Zabalbeitia et al., 2014; Reubold et al., 2015). The DLPs Mitofusin and FZO mediate fusion of the mitochondrial outer membrane, whereas OPA1 and Mgm1 are involved in fusion of the mitochondrial inner membrane system (Hermann et al., 1998; Santel and Fuller, 2001; Frezza et al., 2006; Meeusen et al., 2006).

As in yeast, *Drosophila* and mammals, DLPs are also present in plants and are currently best characterized in *Arabidopsis thaliana*. The DLP ARC5 is localized at the outer membrane of chloroplasts and is involved in chloroplast division (Gao et al., 2003), whereas FZL was found at the stromal side of the chloroplasts envelope and the TM (Gao et al., 2006). This issue makes FZL unique, because it is the only plant DLP within chloroplasts. Potential chloroplast DLPs, their subcellular localizations and potential activities are introduced and further discussed in more detail below (see section “DLPs in *Arabidopsis thaliana*”).

While DLPs are also predicted to exist in prokaryotes, this protein family was ignored for a long time in these organisms. About 10 years ago, the structure and (*in vitro*) activity of the first DLP was described, and the protein of the cyanobacterium *Nostoc punctiforme* was named “bacterial dynamin-like protein” (BDLP, hereafter named *Nos*DLP) (Low and Löwe, 2006). While the exact *in vivo* function of this protein is still to be resolved, *Nos*DLP behaves like other DLPs *in vitro* (Low et al., 2009; Bramkamp, 2012). While DLPs are rather common in cyanobacteria and some species encode multiple DLPs (as further outlined below), further representatives are not experimentally studied yet. Recently, two additional BDLPs were identified and partly analyzed. DynA of *Bacillus subtilis* can mediate membrane fusion *in vitro* (Bürmann et al., 2011), and as there is no obvious need for membrane fusion processes in *B. subtilis*, the *in vivo* activity of DynA was proposed to involve repair of disordered membranes (de Sousa Borges and Scheffers, 2016; Sawant et al., 2016). It was suggested that environmental stress (e.g., induced by phage infection or antibiotics) results in membrane pore formation, and DynA is recruited to these stressed membrane regions where it oligomerizes and fuses opposite bilayer patches in order to seal the membrane (Sawant et al., 2016). Furthermore, the *Escherichia coli* DLP LeoA was suggested to be involved in secretion of toxin-containing vesicles (Brown and Hardwidge, 2007; Michie et al., 2014). More recently two new BDLPs, DynA and DynB, were described to play a key role in a multiprotein cell division complex in *Streptomyces venezuelae* (Schlimpert et al., 2017). DynB is anchored to the cytoplasmic membrane where it interacts with DynA. Both BDLPs colocalize with the tubulin-like GTPase FtsZ and they might be involved, together with additional proteins, in the formation of sporulation septa during cell division (Schlimpert et al., 2017).

All thus far analyzed BDLPs hydrolyze GTP with much lower rates than eukaryotic DLPs (LeoA does not show any GTPase activity) and their GTP hydrolysis rates are typically not affected by lipids (Low and Löwe, 2006; Bürmann et al., 2011; Michie et al., 2014). However, being characteristic for DLPs, *Nos*DLP homodimerizes in its GDP-bound state via its GTPase domain, and in presence of GTP and lipids, the protein self-assembles around liposomes and forms lipid tubes (Low and Löwe, 2006). The *B. subtilis* DynA is an internally fused protein containing two BDLP subunits, and thus, this protein works per definition as a dimer. Consequently, it shows nucleotide independent self-assembly on liposomes (Bürmann et al., 2011). As for LeoA, homo-dimerization has not been shown, suggesting that activation via heterodimerization with LeoBC is crucial (Michie et al., 2014).

Besides these few BDLPs being studied to some extent, the physiological function of DLPs is not clarified in bacteria yet. It is interesting to notice that in chloroplasts and cyanobacteria, both having similar internal membrane systems, membrane dynamics are observed (as discussed above), albeit no clear machinery is yet defined mediating membrane remodeling. Thus, it is not unlikely that BDLPs are involved in membrane dynamics in both chloroplasts and cyanobacteria.

## Common Structural Elements and Domains of (B)DLPs

**FIGURE 2:**
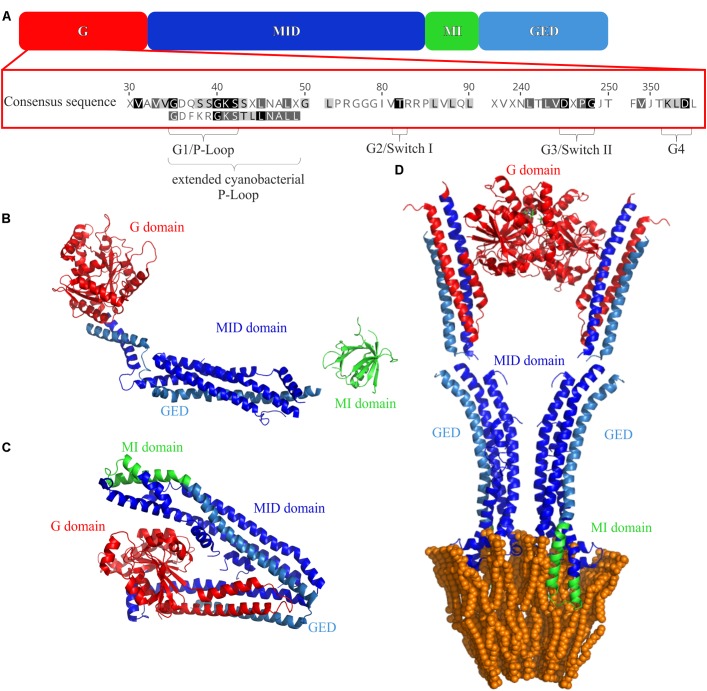
Domain structures of (B)DLPs. **(A)** The G domain carries the GTPase activity, the MID domain is crucial for protein oligomerization, the membrane binding MI domain and the GED are also involved in protein oligomerization. The crystal structures of **(B)** monomeric Dyn G397D ΔPRD (PDB 3ZVR) (Ford et al., 2011), **(C)** *Nos*DLP in its GDP-bound closed monomeric form (PDB 2J69) (Low and Löwe, 2006), and **(D)** an open *Nos*DLP dimer (GDP-bound) anchored in a tubulated lipid bilayer (PDB 2W6D) (Low et al., 2009). The G-domain is colored red, the MID domain in blue and the GED in light blue. The MI domains are highlighted in green.

All (B)DLPs have a very similar domain structure (**Figure 2A**). Typically, a (B)DLP has a G-domain with the GTPase activity, followed by a middle (MID) domain, a region needed for membrane interaction (MI domain) and a GTPase effector domain (GED). The structure of all DLPs is dominated by α-helices, and individual helices often form helical bundle structures. The only highly conserved sequence of (B)DLPs is the G-domain, which is usually located at a (B)DLP N-terminus (**Figure 2A**). It harbors the GTPase domain and contains the amino acid motifs highly conserved in (B)DLPs. Like other GTPases, all (B)DLP share four motifs in the GTP binding/hydrolysis side: G1 or the P-Loop (GxxxxGKS/T), G2 or Switch I (T/S), G3 or Switch II (DxxG), and G4 (RD or N/TxxD). The G1 motif is involved in β-phosphate and Mg^2+^-binding. While the G2 and G3 (D) motifs are also involved in Mg^2+^-binding, they additionally interact with the γ-phosphate of GTP. The G4 motif is also involved in GTP binding but less conserved in (B)DLPs.

In contrast to the G-domain, the remaining (B)DLP domains can vary strongly in their amino acid sequence, but are similar in their function. Often, these domains cannot be identified by sequence comparison and/or homology searches but solely by functional studies.

The MID domain is structurally defined by the presence of α-helical bundles (**Figure 2**), which are crucial for mediating oligomerization of (B)DLPs (Low and Löwe, 2006; Low et al., 2009; Gao et al., 2010; Faelber et al., 2013; Fröhlich et al., 2013; Reubold et al., 2015).

Since (B)DLPs are membrane-active proteins, they harbor one or more MI domains that interact with biological membranes, and these MI domains usually follow the MID domain. However, while membrane interaction is a key feature of DLPs and the basis for membrane remodeling, the exact mode of membrane interaction is not conserved, and different (B)DLPs interact differently with membranes. FZO/Mitofusin, OPA1/Mgm1 as well as Atlastin are anchored to membranes via transmembrane helices. In contrast, the prototypical Dyn interacts with membrane surfaces via the pleckstrin homology (PH) domain, which binds specifically to phosphatidylinositol lipids (Zheng et al., 1996). The DLP MxA harbors a disordered membrane binding loop (L4), and Dnm1 or the yeast homolog Drp1 uses the B insert loop that binds specifically to cardiolipin-enriched bilayers (Mitchell et al., 2013; Bustillo-Zabalbeitia et al., 2014). Furthermore, in EHD2, a polybasic motif was shown to mediate membrane interaction, and in GBP1, a specific Cys residue can be enzymatically isoprenylated, resulting in membrane anchoring (Nantais et al., 1996; Daumke et al., 2007; Vestal and Jeyaratnam, 2011). In case of DynA and *Nos*DLP, the membrane interacting domain is called *paddle domain* (P), and it is named Tip in case of LeoA. The P/Tip domain is dominated by hydrophobic amino acids that mediate interactions with membrane surfaces (Low and Löwe, 2006; Low et al., 2009; Bürmann et al., 2011; Michie et al., 2014).

With only a few exceptions (GBP1 and Atlastin), the GTPase effector domain (GED) follows the MI domain (**Figure 2**). The GED is also part of a helical bundle, and in Dyn, MxA and *Nos*DLP, the GED is additionally involved in the formation of higher-ordered structures (Schumacher and Staeheli, 1998; Chappie et al., 2009; Low et al., 2009).

Furthermore, besides the above described domains, additional domains might exist with more specialized functions.

In summary, all (B)DLPs appear to share three key features: (i) All (B)DLPs oligomerize and form higher ordered structures (Daumke and Praefcke, 2016). While in case of eukaryotic DLPs homo-oligomerization controls the GTPase activity, heterodimerization might be crucial for the activity of BDLPs (Bramkamp, 2012; Michie et al., 2014). (ii) All (B)DLPs are membrane-active and are involved in remodeling nearly every kind of cellular membrane system (reviewed in Bramkamp, 2012; Antonny et al., 2016; Daumke and Praefcke, 2016). For several DLPs it has been shown that they oligomerize *in vitro* and form helical structures around liposomes in the presence of a non-hydrolysable GTP analog, resulting in formation of tube-like membrane structures (Hinshaw, 2000; Low and Löwe, 2006; Bürmann et al., 2011; Mears et al., 2011; Shah et al., 2014; Ugarte-Uribe et al., 2014). (iii) (B)DLPs display a high sequence variability. Besides the G-domain, other domains can typically not be easily predicted in new classes of (B)DLPs and must be experimentally identified.

## DLPs in *Arabidopsis thaliana*

In the model plant *A. thaliana*, 16 DLPs (or DRPs) are encoded. Based on their amino acid sequence and domain structure, these proteins can be grouped in six subfamilies, DRP1-DRP6 (Hong et al., 2003; Backues et al., 2010; Bednarek and Backues, 2010). When using the dynamin signature domain DYN1 (PF00350) for identification of DRPs in *A. thaliana*, combined with the literature and the plant subcellular localization integrative predictor (PSI^1^) (Liu et al., 2013), six DRPs are identified with a putative chloroplast localization. Five of these are designated DRPs in the literature; AtDRP1a/AtADL1a (At5g42080), AtDRP3a/AtADL2a (At4g33650), AtDRP3b/AtADL2b (At2g14120), AtDRP5A (At1g53140), and AtDRP5B/AtARC5 (At3g19720), whereas one is named fuzzy onion (FZO)-like protein (FZL). AtDRP1a/AtADL1a, AtDRP3a/AtADL2a, and AtDRP3b/AtADL2b all contain the GTPase (DYN1, PF00350), the dynamin MID region (DYN2, PF01031) and the GTPase-effector domain (GED, PF02212) (Miyagishima et al., 2008; Heymann and Hinshaw, 2009). The proteins AtDRP5A and AtDRP5B/AtARC5 additionally contain a pleckstrin homology domain (PH, PF00169) that binds to membrane phospholipids. In contrast, the FZL protein (At1g03160) contains solely the DYN1 signature domain.

AtDRP1a/AtADL1a is one of five proteins in the DRP1 subfamily. AtDRP1a/AtADL1a was found in TMs and was suggested to be involved in vesicle formation inside chloroplasts due to impaired chloroplast development including reduced amount of chloroplast membranes (Park et al., 1998). However, AtDRP1a/AtADL1a was also identified at the cell plate (Lauber et al., 1997). This apparent discrepancy was explained by a shortcoming of the antibody used in the study of Park et al. (1997). While the antibody was expected to recognize the GTPase domain of AtDRP1a/AtADL1a, the GTPase domains of DLPs are generally highly conserved (as discussed above), and thus the antibody could well have detected other DLPs besides AtDRP1a/AtADL1a (Kang et al., 2001). Subsequent studies have further challenged the assumption of chloroplast localization, as AtDRP1a/AtADL1a is targeted to other cellular compartments and was shown to have other roles (Kang et al., 1998, 2003; Collings et al., 2008; Konopka and Bednarek, 2008; Fujimoto et al., 2010; Yoshinari et al., 2016). The protein is targeted to the cell plate during cytokinesis (Kang et al., 1998), and in mutants lacking AtDRP1a/AtADL1a, an unusual plasma membrane accumulation is observed. This seems to inhibit efficient targeting and fusion of exocytic vesicles to the cell surface, which disturbs cell wall production (Kang et al., 2003). Moreover, the protein has also been shown to have a role in endocytic events at the plasma membrane, possibly associated with formation of clathrin-coated vesicles (Collings et al., 2008; Konopka and Bednarek, 2008; Fujimoto et al., 2010; Yoshinari et al., 2016). Thus, AtDRP1 is currently not considered to be active in chloroplasts.

AtDRP3a/AtADL2a has also been predicted to be localized in chloroplasts. A GFP-tagged version of the *A. thaliana* DRP3a has been shown to be chloroplast-localized in soybean and tobacco, where the N-terminal 35 amino acid residues were shown to be sufficient for chloroplast targeting (Kang et al., 1998). However, in a later study a GFP-DRP3a fusion protein was observed in mitochondria rather than in chloroplasts and was shown to be involved in mitochondrial division (Arimura et al., 2004). Moreover, AtDRP3 has also been partially targeted to peroxisomes where it has been suggested to have an essential role in peroxisome fission and replication (Lingard et al., 2008; Zhang and Hu, 2009; Mano et al., 2011). Thus, although an AtDRP3 mutant displays slow growth and a pale color at the seedling stage (Zhang and Hu, 2009), AtDRP3a less likely functions inside chloroplasts, but instead the protein localizes to mitochondria and peroxisomes.

AtDRP3b, also known as “Arabidopsis dynamin-like 2b” (ADL2b), shares 76% sequence identity with AtDRP3a/AtADL2a (Hong et al., 2003). While the protein is predicted (by the plant subcellular tool PSI) to be chloroplast-localized, is has so far not been shown to be targeted to chloroplasts. Instead, it has been identified in mitochondria and peroxisomes where it is suggested to support mitochondrial and peroxisomal division, respectively (Arimura and Tsutsumi, 2002; Fujimoto et al., 2009; Zhang and Hu, 2009). The phenotype of a mutant is similar to a AtDRP3a mutant, i.e., retarded growth and pale colored at the seedling stage (Zhang and Hu, 2009). While both, AtDRP3a/AtADL2a and AtDRP3b/AtADL2b, are suggested to be involved in mitochondrial as well as in peroxisomal division, in mitochondria the proteins have redundant functions while in peroxisomes they appear to have more distinct functions (Fujimoto et al., 2009).

Similar to AtDRP3b, the PSI tool predicts the DRP5 subfamily member AtDRP5a to be targeted to chloroplasts, although this localization has yet to be confirmed experimentally. The phenotype of mutant plants shows retarded seedling growth with no altered chloroplast (Miyagishima et al., 2008). Thus, there is currently no clear link to chloroplasts except for a putative localization prediction. Instead, AtDRP5a has been identified via GFP-tagging and immunoblot analyses in the cytosol of meristematic and meristemoid cells, and the protein is mainly found within dividing cells and suggested to be involved in cytokinesis (Miyagishima et al., 2008).

AtDRP5b is localized in both chloroplasts and peroxisomes, and mutant plants show retarded plant growth with yellowish leaves and enlarged and dumbbell-shaped chloroplasts (Gao et al., 2003; Zhang and Hu, 2010). AtDRP5b, also known as “accumulation and replication of chloroplasts 5” (ARC5), has no predicted signal sequence-mediating protein import into chloroplasts, albeit it clearly localizes to this organelle. However, AtDRP5b/ARC5 is found at the outer chloroplasts envelope membrane facing the cytosol, where it is enrolled in division ring construction at the late stage of the chloroplast division (Pyke and Leech, 1994; Gao et al., 2003; Miyagishima et al., 2006). The protein is recruited to the plastid division site by two plastid division proteins (PDV1 and PDV2), which also regulate the GTPase activity of AtDRP5b/ARC5 (Gao et al., 2013; Holtsmark et al., 2013). However, AtDRP5b is also present in peroxisomes as revealed by bimolecular fluorescence complementation and co-immunoprecipitation assays, and when the AtDRP5b gene was mutated, impaired peroxisome division and function was observed (Zhang and Hu, 2010).

The *A. thaliana* protein AtFZL is related to fuzzy onion (FZO) proteins that are part of the dynamin superfamily of remodeling GTPases. FZO is a protein that is located in the outer mitochondria membrane where it is involved in fusion of opposing outer mitochondrial membranes in animals and fungi (Koshiba, 2004; Meeusen, 2004). However, the *A. thaliana* FZL protein shows low homology both to the Mitofusin domain found in the FZO family as well as to the dynamin domain (DYN1). Despite some sequence homology and similarities with FZO in respect of existing domains and their arrangement (GTPase, coiled-coil, transmembrane helices), absence of AtFZL does not affect mitochondria morphology in *A. thaliana* but instead the morphology of chloroplasts (Gao et al., 2006). AtFZL is located inside the chloroplasts at the TM but also at the chloroplast inner envelope (Gao et al., 2006). It is believed to be anchored to these membranes via two transmembrane domains located within the C-terminal part of the protein, leaving the GTPase and coiled-coil domains protruding into the chloroplast stroma (Gao et al., 2006). Whether the AtFZL operates in a similar fashion as the classical FZO, i.e., whether it brings two membranes into close contact resulting in membrane fusion, is currently unknown. However, such an activity is indicated and an involvement of AtFZL in the transport of lipids between the inner envelope and the TM has been suggested, since vesicles appear to not fuse in plants lacking AtFZL (Gao et al., 2006) and since the chloroplasts show a disorganized TM morphology. Thus, AtFZL likely is a membrane-remodeling GTPase, involved in TM biogenesis and dynamics in chloroplasts (Gao et al., 2006).

Thus, out of the six predicted DRPs, evidence for chloroplast localization and function is only strong for AtDRP5b and AtFZL. However, as AtDRP5b is localized at the outer envelope membrane, AtFZL is the one remaining that could facilitate membrane remodeling, potentially involving fission and fusion of vesicles budding off from the inner envelope membrane and being targeted to the TM (Kroll et al., 2001; Wang et al., 2004; Garcia et al., 2010; Tanz et al., 2012; Armbruster et al., 2013; Karim and Aronsson, 2014; Karim et al., 2014). Thus, DRPs can be important to secure a dynamic but organized thylakoid network in chloroplasts.

## DLPs Are Conserved in Cyanobacteria

The BDLP of the cyanobacterium *N. punctiforme* has been characterized to some extent. As typical for DLPs, the smallest structural unit of this BDLP appears to be a dimer, which can further self-assemble (Low and Löwe, 2006; Low et al., 2009). *Nos*DLP is thought to mediate membrane fusion by inducing formation of highly curved membrane regions (Low and Löwe, 2006). After GTP-binding, *Nos*DLP self-oligomerizes *in vitro* around a membrane and forces the membrane into a tube-like structure with high curvature, similar to the prototypical eukaryotic Dyn protein that is involved in vesicle fission. After GTP hydrolyzes, *Nos*DLP is released from the lipid, and adjacent membrane regions spontaneously fuse (Low et al., 2009). A *Nos*DLP-GFP fusion protein was found in the cell periphery in *N. punctiforme* as well as in rare ring-like structures at the cell septa (Low and Löwe, 2006), highlighting that the protein is interacting with membranes and is membrane-active. Nevertheless, the *in vivo* function of this protein is still unclear.

**FIGURE 3:**
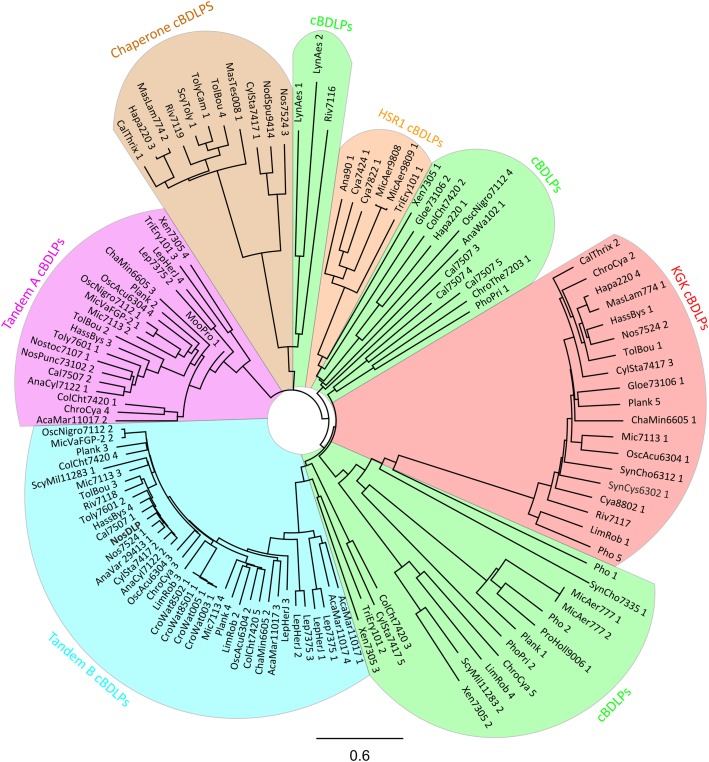
Phylogenetic tree of 121 cBDLPs encoded in 56 cyanobacteria species (including *Nos*DLP). The DLP discussed in the text is shown in bold. The name of the potential dynamins is abbreviated. For full information about the strain, the gene name and the gene locus, view the **Supplementary Data Sheet S1**. The cBDLPs can be classified into six different clades, depending on their sequence and genomic environment: the *KGK* clade (red), the *HSR1* clade (orange), and the *chaperone* clade (brown). Upstream of the *Tandem A* (pink) clade, another cBDLP (*Tandem B*, light blue) is encoded. cBDLPs (green) could not be classified further and thus, represents a group of diverse cBDLPs. It is worth mentioning that boundaries between the *cBDLPs* clade and the *Tandem B* or *HSR1* clades, respectively, are not sharp and it might be that proteins share characteristics of both clades. Furthermore, while Xen7305 3 does not show significant sequence similarity to Tandem B members, downstream of this gene, the Tandem A protein Xen7305 4 is encoded. The here described group characteristics refer to the majority of the cBDLPs but do not represent every encoded protein. For further details, view the **Supplementary Data Sheet S1**. The phylogenetic tree (model: jukes-cantor, neighbor-joining) was created by a full-length multiprotein sequence alignment (Geneious global alignment, Matrix Blosum62) implemented in the software Geneious version 11.0.4 (http://www.geneious.com; Kearse et al., 2012). The cyanobacterial sequences were obtained from “cyanobase” (Nakamura et al., 1998; Fujisawa et al., 2017).

To obtain further information about potential cyanobacterial BDLPs (hereafter named cBDLPs to distinguish them from other bacteria DLPs), we searched the *pfam* database (dynamin_N family PF00350), for cyanobacterial proteins carrying a dynamin GTPase domain, the only key marker for DLPs. Based on this analysis, 279 potential cBDLPs were identified being encoded in 74 different cyanobacterial species. However, we further limited our results and removed small GTPases, which sometimes also have a predicted dynamin GTPase domain. Finally, we ended up with 121 genes that likely encode BDLPs in 56 different cyanobacterial strains (**Figure 3** and **Supplementary Data Sheet S1**). It should be noted that due to the rather rigid search, we might have overlooked some cBDLPs. While all potential cBDLPs have a highly conserved dynamin-like GTPase domain within the N-terminal protein region, we did not limit our search to the identification of other known domains, since the sequences of these domains are typically not conserved (as discussed above). However, nearly all of the cBDLP sequences have an extended and conserved P-loop region within the G-domain: beside the common GxxxxGKS/T P-loop motif, cBDLPs have an additional L/INALL/I motif, which extends the P-loop motif to GxxxxGKS/TxL/INALL/I.

Unfortunately, none of the predicted cDLPs share a high sequence identity with the AtFZL protein in chloroplasts. However, based on a phylogenetic analysis we categorized the identified cBDLPs into six groups, where the sequences of individual cBDLPs are highly conserved within the defined clades but clearly differ in between the clades (**Figure 3**). Interestingly, the genetic context of some cBDLP is conserved in some clades and proteins are, e.g., part of conserved gene clusters (**Supplementary Data Sheet S1**).

Within the first cBDLP group, typically a KGK domain protein is encoded downstream of the cBDLP. Members of the KGK protein family (PF08872) are small cyanobacterial proteins (around 120 amino acids) that contain a KGK domain (Finn et al., 2014). Unfortunately, the precise function of this domain is enigmatic, albeit this domain potentially mediates protein–protein interactions. The second cBDLP group is termed *Tandem B* cBDLPs, and the only yet characterized cBLPD, the BDLP of *N. punctiforme*, belongs to this group (Low and Löwe, 2006). Upstream of the encoding gene, a second cBDLP is encoded (clade *Tandem A*), and thus, translation of the clustered genes likely results in expression of two different BDLPs. While it has been shown that *Nos*DLP forms homodimers, its GTPase activity was not at all affected by homo-oligomerization and/or lipid binding. Therefore, it has already been suggested that hetero-oligomerization with another, different BDLP might control the activity of this BDLP in a way, as observed in the case of eukaryotic DLPs (DeVay et al., 2009; Michie et al., 2014). Based on our analyses, the Tandem A representative of *N. punctiforme* is a likely candidate. Noteworthy, the sequences of the Tandem A and Tandem B cBDLPs differ substantially.

In the *HSR1 group* of cBDLPs, a potential protein of the HSR1 protein family is encoded downstream of the cBDLP. HSR1-releated proteins are not well-characterized GTP-binding proteins that, however, have no apparent GTPase enzymatic function (Finn et al., 2017). Thus, the cyanobacterial HSR1 proteins encoded adjacent of the cBDLPs potentially have a regulatory function. In the *chaperone group*, conserved proteins carrying a DnaK domain are encoded downstream of the respective cBLDP. DnaK proteins belong to the group of Hsp70 chaperones (Mayer and Bukau, 2005; Young, 2010; Mayer, 2013), and thus, here the activity of a membrane remodeling DLP is linked to the activity of an Hsp70 chaperone. In fact, membrane activity of Hsp70 members has been described in recent years (Armijo et al., 2014; Mahalka et al., 2014). In humans, selected Hsp70 proteins have been suggested to, e.g., carry immunogenic peptides for antigen presentation (Haug et al., 2005), and thus, DnaK-like proteins encoded in the vicinity of a cBDLP might be involved in protein sorting during membrane remodeling processes.

The remaining cBDLPs are not significantly related to one another and could not be further categorized (named *cBDLP* in **Figure 3**).

## Are (B)DLPs Involved in TM Biogenesis and Dynamics in Chloroplast and Cyanobacteria?

At least one DLP is present in chloroplasts and the activity of the FZL protein has been linked to membrane biogenesis and dynamics (Gao et al., 2006). As discussed before, DLPs are also encoded in cyanobacteria and at least the *Nos*DLP can remodel membranes resulting in membrane fission (Low et al., 2009). However, the exact *in vivo* function of (B)DLPs in chloroplasts and cyanobacteria still is enigmatic. Clearly, membrane disruption would be deleterious in chloroplasts as well as in cyanobacteria, e.g., disruption of TMs would result in a breakdown of the electrochemical gradient across the TM. Thus, it is feasible to propose a membrane protective function of the DLPs and a crucial role in the repair of ruptured membrane regions, as suggested, e.g., for the BDLP of *B. subtilis* (Sawant et al., 2016). However, in cyanobacteria different proteins and systems are described that are suggested to be involved in membrane stabilization and membrane repair, involving small heat shock proteins and the PspA system (Torok et al., 2001; Nitta et al., 2005; Manganelli and Gennaro, 2017). Thus, the DLPs might have acquired additional functions in chloroplasts and cyanobacteria.

**FIGURE 4:**
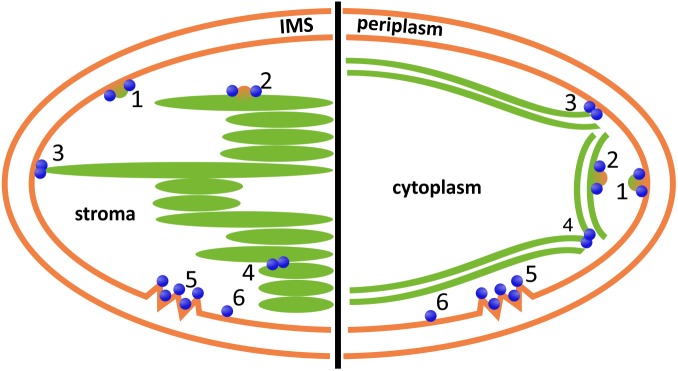
Potential involvement of DLPs in chloroplast **(left)** and cyanobacterial **(right)** inner membrane dynamics. DLPs might be involved in vesicle formation, release or fusion at the chloroplast inner envelope or the cyanobacterial cytoplasmic membrane, respectively (1), or at the TMs (2); the fusion of the inner envelope or cytoplasmic membrane, respectively, with the TM system (3); and/or the fusion/constriction of individual TM layers (4). Moreover, DLPs are potentially involved in membrane repair (5) and/or membrane protection (6). IMS, inter membrane space. Note that DLPs located outside of the chloroplast, involving AtDRP5B/ARC5, are excluded.

In recent years, evidence has accumulated indicating that membrane fusion and fission events are involved in TM development and dynamics in chloroplasts and cyanobacteria (Chuartzman et al., 2008; Kirchhoff et al., 2011; Herbstova et al., 2012; Nevo et al., 2012; Iwai et al., 2015). As spontaneous, uncontrolled membrane fusion would be deleterious to organisms, defined fusion and fission machineries likely control such remodeling processes (Chuartzman et al., 2008). However, proteins involved in membrane dynamics in chloroplast and cyanobacteria still need to be better characterized and more need to be identified. In bioinformatic analyses, several genes have been identified that code for putative chloroplast-localized proteins with homology to proteins involved in the secretory pathway, operating in the cytoplasm of eukaryotic cells, and some of these proteins are also conserved in cyanobacterial genomes (Nakai et al., 1993; Srivastava et al., 2005; Keller and Schneider, 2013; Khan et al., 2013; Paul et al., 2014). Nevertheless, potential involvement of these proteins in TM biogenesis and/or maintenance has only sparsely been shown experimentally yet, and it appears to be rather unlikely that vesicle fission and fusion is regulated identical in chloroplasts as in the secretory pathway. However, some of the identified (putative) membrane-active proteins might fulfill similar functions in chloroplasts as in the secretory pathway but work together with other, chloroplast- and cyanobacteria-specific proteins. Such chloroplast and cyanobacteria-specific proteins likely involve the recently identified Vipp1/IM30 protein (Kroll et al., 2001; Westphal et al., 2001), a protein that can fuse membranes in presence of Mg^2+^, at least *in vitro* (Hennig et al., 2015). Moreover, IM30-depleted chloroplasts and cyanobacteria have a significantly reduced TM network (Kroll et al., 2001; Fuhrmann et al., 2009). Within the secretory pathway, several small GTPases are involved in vesicle formation and fission (Hutagalung and Novick, 2011; Barlowe and Miller, 2013), and involvement of the small GTPases AR1 and CPRabA5e in TM biogenesis has been shown in *A. thaliana* (Garcia et al., 2010; Karim et al., 2014). Since especially dynamin-like GTPases are directly associated with membrane remodeling processes in many eukaryotic organelles, it appears possible that DLPs are also involved in membrane biogenesis and/or remodeling processes in chloroplasts and cyanobacteria. In fact, FZL has been suggested to mediate contact of two adjacent membranes in *A. thaliana* chloroplasts, finally resulting in membrane fusion (Gao et al., 2006). The here presented analysis clearly demonstrates that DLPs are also highly abundant in cyanobacteria. However, thus far solely the DLP of the cyanobacterium *N. punctiforme* has been studied to some extent. The *in vitro* analyses clearly indicate that the protein behaves like the classical Dyn and thus might be involved in vesicle fission in cyanobacteria. Nevertheless, we initially expected to identify a prototypical cDLP in our analysis that is conserved in all cyanobacterial species. Surprisingly, we did not identify such a candidate; rather, while most cyanobacterial genomes encode at least one cDLP, the proteins belong to different clades. Thus, the sequences of cBDLPs are highly variable. While the exact physiological function of the cyanobacterial proteins is enigmatic, it is reasonable to assume that proteins with a membrane remodeling activity will be involved in membrane dynamics in chloroplasts and cyanobacteria. The exact physiological function of the proteins, i.e., their involvement in processes such as membrane protection, membrane repair, membrane fission and/or membrane fusion, however, still needs to be established. Nevertheless, the *in vivo* observation of FZL being involved in TM dynamics and vesicle fusion and the *in vitro* observation of *Nos*DLP behaving like the prototypical Dyn clearly indicates a crucial membrane-active role of DLPs in chloroplasts and cyanobacteria. Based on the described membrane activities of DLPs and on the need of membrane remodeling processes, in **Figure 4** we summarize a potential involvement of DLPs in chloroplasts and cyanobacteria. We hope this article will stimulate future research on the involvement of this membrane-active protein family in membrane dynamics in chloroplasts and cyanobacteria.

## Author Contributions

All authors listed have made a substantial, direct and intellectual contribution to the work and approved it for publication.

## Conflict of Interest Statement

The authors declare that the research was conducted in the absence of any commercial or financial relationships that could be construed as a potential conflict of interest.

## Footnotes

1 http://bis.zju.edu.cn/psi/

## Supplementary Material

**DATA SHEET S1 |** Cyanobacterial species.
